# Quality of Information on Lip Tie in YouTube Videos: A Content Analysis

**DOI:** 10.7759/cureus.73005

**Published:** 2024-11-04

**Authors:** Maya Raghavan, Erin M Gawel, Arunima Vijay, Michele M Carr

**Affiliations:** 1 Otolaryngology, Jacobs School of Medicine and Biomedical Sciences, Buffalo, USA; 2 Otolaryngology, University of Florida College of Medicine, Gainesville, USA

**Keywords:** labial frenulum, lip tie, pediatric otolaryngology, quality assessment, social media

## Abstract

Objective

"Lip tie" is a term that has become commonly used to refer to a prominent or short maxillary frenulum and is controversially associated with difficulties in breastfeeding. There has been a rise in the popularity of lip tie division without clear expert agreement on the indications and benefits of treatment. Our study aims to determine the quality of information on YouTube about lip ties.

Methods

A YouTube search for "lip tie" was performed in February 2023, and the top 50 English language results were viewed and evaluated. Viewer interest parameters, such as the number of video views, likes, and page subscribers, were recorded at the time of viewing. Information about video content was also recorded. The included videos were analyzed by two independent reviewers for quality using modified DISCERN criteria and Journal of the American Medical Association (JAMA) scoring.

Results

There was a median of 12779 (IQR: 3129-31318) views, 86 (IQR: 17-262) likes, and 1950 (IQR: 462-38300) subscribers. Videos were available online for a mean of 3.72 years. Video authors consisted of 21 (42%) dentists, six (12%) United States (US)-based otolaryngologists, 12 (24%) YouTube influencers, four (8%) US-based MD/DO non-otolaryngologists, four (8%) lactation consultants, two (4%) laser companies, two (4%) myo-functional therapists, and one (2%) chiropractor. The total mean modified DISCERN score was 2.2 ± 1.0 out of 5, and the mean JAMA score was 1.8 ± 0.5 out of 4. Otolaryngologists had the highest modified DISCERN score, followed by dentists and non-otolaryngologist physicians. Non-otolaryngologist physicians had the highest mean JAMA score. Educational videos had a significantly higher mean modified DISCERN score than testimonials (p=0.007).

Conclusions

When parents refer to YouTube for information about lip ties, search results yield low-quality information overall. Most videos are from dentists and YouTube influencers in support of frenotomy, and this information may lead parents to seek unnecessary surgery for their children.

## Introduction

In the past 20 years, literature correlating a thick or prominent maxillary labial frenulum with negative consequences on infant development has increased by 700% [1]. The maxillary labial frenulum comprises alveolar mucosal tissue that attaches the upper lip to the maxillary gingiva. When it is especially thick, prominent, or perceived to be causing problems with development, it is referred to as a "lip tie" [2]. Literature about lip ties focuses on improper latching during breastfeeding and poor oral hygiene due to a perceived decrease in upper lip mobility; however, the evidence remains contradictory and controversial [3]. Growing public awareness of lip ties has led to concern among parents and an increase in providers offering corrective procedures, despite the lack of consensus on indications for the procedure and the best practices for intervention [4].

Approaches to managing lip ties are drawn from the better-established guidelines for correcting ankyloglossia or "tongue ties" [3]. Still, there are no clinical guidelines specifically associating the anatomical presentation of a lip tie with the likelihood of sequelae. Currently, the Kotlow and Stanford systems are used to classify lip ties based on the anatomy of the maxillary frenulum, but evidence suggests that the degree of classification by these systems is not related to the severity of symptoms in infants [3,5].

The lack of clear guidelines for lip tie management and varying advice from healthcare professionals has led parents to seek alternative online resources to guide their clinical decision-making [6-8]. Platforms like YouTube are particularly popular as they enable parents to form socially and emotionally supportive communities to share information regardless of geographical barriers. However, platforms like YouTube are also susceptible to spreading misinformation due to their open-source format [9].

This study aims to analyze the content and quality of information pertaining to lip ties on YouTube as a platform that may influence parent education and clinical decision-making for lip tie management.

## Materials and methods

An observational study was conducted using public data on YouTube. YouTube was searched using the keyword "lip tie" on March 4-5, 2023, for videos published at any time. The Incognito setting was used to limit bias from specific user account preferences. The first 50 English-language videos related to lip ties, ranked by relevance by YouTube's algorithm, were included in this study. Videos containing information only related to tongue ties were excluded. Videos duplicated in part were included to best represent the top videos that YouTube users were most likely to watch. The study was exempt from approval by the University at Buffalo Institutional Review Board due to the use of publicly accessible data. There were no human or animal participants, and the authors did not have access to data that could identify individuals.

Fifty videos accounted for approximately the first three pages of search results. Studies have shown that 92% of users typically click on a result within the first three pages, with 68% of users clicking a result on the first page [10]. If the desired information is not found within the first three pages, users are more likely to change their search terms than to continue looking through the initial search results [11].

Each video was viewed and analyzed by two independent reviewers. Viewer interest parameters, including the number of video views, likes, comments, and page subscribers, were recorded at the time of viewing. Information regarding video content was also noted, and video inclusion of lip tie definition, clinical features, and treatment were analyzed. Videos were categorized into six groups by source: independent users, otolaryngologists, non-otolaryngologist physicians, dentists, other health professionals, and surgical laser companies.

Quality assessment

Included videos were analyzed using both a five-item questionnaire modified from the DISCERN instrument (Table 1) and a four-item questionnaire from the Journal of the American Medical Association (JAMA) scoring system (Table 2). The modified DISCERN scoring system includes questions related to publication reliability and quality [11,12]. JAMA criteria address authorship, attribution, disclosure, and currency of content. Higher scores on each scale are indicative of increased content reliability [13].

**Table 1:** Modified DISCERN scoring system

Item	Description
1	Are the aims clear and achieved?
2	Is the information presented balanced and unbiased?
3	Are reliable sources of information used?
4	Does it provide additional sources of support and information?
5	Does it refer to areas of uncertainty?

**Table 2:** Journal of the American Medical Association (JAMA) quality assessment

Criteria	Description
Authorship	Are authors and contributors, their affiliations, and relevant credentials provided?
Attribution	Are references and sources listed clearly, and relevant copyright information noted?
Disclosure	Are conflicts of interest, funding, sponsorship, advertising, support and video ownership disclosed?
Currency	Is the initial date of posted content indicated?

Statistical analysis

Statistical analysis was conducted using Stata 15 (StataCorp LLC, College Station, TX). Categorical variables were summarized as frequency counts and percentages. Continuous variables were summarized as mean ± standard deviation (SD) or median and interquartile range (IQR). Kruskal-Wallis was performed to compare non-parametric variable medians across categories. Linear regression models were built using backward stepwise regression. A p-value <0.05 was considered significant.

## Results

Fifty videos were included with a median of 12779 (IQR: 3129-31318) views, 86 (IQR: 17-262) likes, and 1950 (IQR: 462-38300) subscribers. Videos had been available online for a mean of 3.72 years. Video sources included 12 (24%) independent users, seven (14%) otolaryngologists, four (8%) non-otolaryngologist physicians, 18 (36%) dentists, five (10%) other health professionals, and four (8%) surgical laser companies. Other health professionals included two lactation consultants, two myo-functional therapists, and one chiropractor. Descriptive data for each video group can be seen in Table 3.

**Table 3:** Distribution of viewer interest parameters by video source ^a^Video comments were turned off. Data presented as mean ± standard deviation (SD).

	Subscribers	Number of views	Likes	Comments
Independent user	84105 ± 156565	22481 ± 26505	255 ± 208	79 ± 97
Otolaryngologist	260149 ± 233090	132047 ± 280577	292 ± 275	29 ± 31
Non-otolaryngologist physician	775231 ± 894626	186516 ± 291403	1199 ± 1727	N/A^a^
Dentist	1394 ± 3601	22676 ± 29946	84 ± 95	8 ± 12
Other health professional	15694 ± 21347	75178 ± 165275	329 ± 710	3 ± 0.7
Surgical laser company	6030 ± 10114	16377 ± 14151	39 ± 28	7 ± 5

Topics most discussed in the YouTube videos are presented in Table 4. Twenty (40%) videos mentioned breastfeeding difficulties as a reason to seek care. Six (12%) videos mentioned that frenotomy is not always effective, and three (6%) videos discussed overdiagnosis or overtreatment of lip ties.

**Table 4:** Topics most frequently discussed in YouTube videos Data presented as N (%).

Topic	N (%)
Lip tie definition	26 (52)
Diagnostic process	33 (64)
Symptoms	29 (58)
Treatment	
Frenotomy	45 (90)
Non-surgical management	16 (32)
Lactation consultant	11 (22)
Chiropractor	3 (6)
Osteopathic evaluation	1 (2)
Occupational therapist	1 (2)

The most commonly mentioned symptoms were fussiness, tooth gap, poor oral hygiene, dribbling while eating, spitting up, unlatching during breastfeeding, clicking sounds with feeding, and frequent feeds (Table 5). The most recommended reasons to seek care were painful breastfeeding and difficulty latching. Testimonial videos, more than any other type, encouraged viewers to seek medical care if they experienced these issues with their infant.

**Table 5:** Symptoms of lip tie discussed in YouTube videos Data presented as N (%).

Symptom	N (%)
Difficulty latching	20 (40)
Restricted lip mobility	18 (36)
Painful breastfeeding	18 (36)
Tooth gap	12 (24)
Tooth decay/poor oral hygiene	11(22)
Colicky/fussy	8 (16)
Spitting up	8 (16)
Poor sleep	5 (10)
Low weight gain	4 (8)

The total mean modified DISCERN score was 2.2 ± 1.0 out of 5, and the mean JAMA score was 1.8 ± 0.5 out of 4. Otolaryngologists had the highest modified DISCERN score, followed by dentists and non-otolaryngologist physicians. Non-otolaryngologist physicians had the highest mean JAMA score (Table 6). Linear regression showed that an increased modified DISCERN score was associated with a decreased number of video comments (p=0.02). The number of video likes and page subscribers was not significantly associated with modified DISCERN or JAMA scores. Educational accounts run by otolaryngologists, other physicians, and dentists had significantly fewer page subscribers than individual users (p=0.001), but there was no statistical difference in number of views by video source. Educational videos had a significantly higher mean modified DISCERN score and JAMA score than testimonials uploaded by independent users (p=0.007 and p<0.001, respectively).

**Table 6:** Mean modified DISCERN score and JAMA score by video source Modified DISCERN score and JAMA score calculations are detailed in Methods. Categorical data presented as N (%) and continuous data presented as mean ± SD.

Source	N (%)	Modified DISCERN score ± SD	JAMA score ± SD
Independent user	12 (24)	1.6 ± 1.0	1.2 ± 0.4
Otolaryngologist	7 (14)	3.1 ± 0.7	2.0 ± 0.0
Non-otolaryngologist physician	4 (8)	2.3 ± 1.3	2.3 ± 0.4
Dentist	18 (36)	2.8 ± 0.7	1.9 ± 0.3
Other health professional	5 (10)	1.5 ± 0.4	2.0 ± 0.0
Surgical laser company	4 (8)	1.0 ± 0.6	1.8 ± 0.5

## Discussion

This study evaluated the content and quality of lip tie information on YouTube to better understand the resources parents are accessing that may impact healthcare decisions for their infants. As alternative health resources become more frequently utilized, physicians and patients alike are impacted by the quality of publicly available information online. To our knowledge, there are currently no published studies evaluating YouTube as a source of patient information on lip ties.

Our investigation found that breastfeeding difficulty was presented as the most common reason a lip tie is diagnosed and treated. Painful breastfeeding and difficulty latching and maintaining the latch were cited as reasons mothers may seek care for themselves and their infants. Current literature supports these findings as the main reasons parents seek specialist care for suspected lip ties [14,15]. YouTube videos also discussed spitting up, poor oral hygiene, and tooth gaps as common consequences of lip ties. These symptoms are also commonly referenced in the literature, but there is insufficient evidence to support lip ties as the definitive cause [16-18].

The potential overdiagnosis and unnecessary surgical intervention for lip ties is a growing concern among otolaryngologists [19]. Only three videos discussed the uncertain association between lip ties and the symptoms mentioned or the fact that frenotomy may not result in symptom improvement. Overall, in YouTube videos, a recommendation for frenotomy was disproportionately higher than for nonsurgical management. Frenotomy was mentioned in 90% of videos and was most often presented as a definitive treatment for lip ties and its reported associated symptoms, whereas only 32% of videos mentioned consulting a lactation specialist for breastfeeding difficulties. No videos discussed observation as a management option.

This data demonstrates that YouTube videos providing lip tie information strongly emphasize surgical revision, even though the current literature does not clearly show a benefit. Despite heightened parental concerns about lip ties, it has been shown that infants with lip ties do not experience feeding difficulties more than the general population [16]. Previous studies have failed to demonstrate a correlation between maxillary frenulum anatomical grade and breastfeeding, pain, or poor latching, making clinical diagnosis difficult [3,16,20]. Although some studies show that frenotomy may improve breastfeeding, not all infants require a frenotomy to resolve feeding difficulties [1,21].

The mean modified DISCERN score and mean JAMA score were low, indicating that the information available is generally of poor quality. Most videos failed to provide sources for their information or to provide further resources for their viewers. Viewers, therefore, cannot easily verify the information in videos or find further trusted sources. Testimonials from independent users had more page subscribers and interactions than educational videos, although educational videos had the same number of views. YouTube users may be discussing their experiences with each other online, but they are still viewing educational videos during their research.

Previous studies have evaluated YouTube as a source of information for functional endoscopic sinus surgery, rhinosinusitis, and cochlear implant surgery [22-24]. Information was found to be highly variable and unreliable. These findings are similar to the results from our study. Our study adds to current evidence that YouTube is a poor source of information about health conditions and surgical procedures, although it continues to be commonly used.

Limitations

This study was limited by a small sample size of videos and the exclusion of non-English language content. This study was cross-sectional and only evaluated YouTube videos at one point in time. Since content is continuously being uploaded onto YouTube, search results change frequently, and the results of this study may not be applicable in the long term. Video results of related search terms such as "labial frenotomy" or "maxillary frenulum" were not included in this analysis because parents would likely search for the term "lip tie." Future studies should evaluate viewer interaction to see how patient perspectives are influenced by YouTube videos on lip ties. Analysis could be expanded to other social media platforms as well.

## Conclusions

The quality of information on lip ties on YouTube is poor and may lead parents to seek unnecessary surgery for their children. There is an absence of data suggesting a causal relationship between lip ties and breastfeeding difficulties, although many parents may request surgical revision based on information found online recommending frenotomy. Physicians can improve patient education by being aware of what parents may be watching online and providing more reliable resources that suggest both appropriate surgical and nonsurgical management options for difficulty breastfeeding.

## References

[1] Towfighi P, Johng SY, Lally MM, Harley EH (2022). A retrospective cohort study of the impact of upper lip tie release on breastfeeding in infants. Breastfeed Med.

[2] Santa Maria C, Aby J, Truong MT, Thakur Y, Rea S, Messner A (2017). The superior labial frenulum in newborns: what is normal?. Glob Pediatr Health.

[3] Shah S, Allen P, Walker R, Rosen-Carole C, McKenna Benoit MK (2021). Upper lip tie: anatomy, effect on breastfeeding, and correlation with ankyloglossia. Laryngoscope.

[4] Fraser L, Benzie S, Montgomery J (2020). Posterior tongue tie and lip tie: a lucrative private industry where the evidence is uncertain. BMJ.

[5] Kotlow LA (2004). Oral diagnosis of abnormal frenum attachments in neonates and infants: evaluation and treatment of the maxillary and lingual frenum using the Erbium: YAG laser. J Pediatr Dent Care.

[6] Bryan MA, Evans Y, Morishita C, Midamba N, Moreno M (2020). Parental perceptions of the internet and social media as a source of pediatric health information. Acad Pediatr.

[7] Ward LM, Sykora CA, Prakash Y, Cohen MB, Levi JR (2022). Parental views on the role of social media in pediatric otolaryngology. Ann Otol Rhinol Laryngol.

[8] AlFraih Y, AlGhamdi M, AlShehri A, AlTokhais T (2023). Social media in pediatric surgery: patient perceptions and utilization. J Pediatr Surg.

[9] Frey E, Bonfiglioli C, Brunner M, Frawley J (2022). Parents’ use of social media as a health information source for their children: a scoping review. Acad Pediatr.

[10] Blandford A (2024). iProspect blended search results study. Dalhous J Interdisci.

[11] Singh AG, Singh S, Singh PP (2012). YouTube for information on rheumatoid arthritis--a wakeup call?. J Rheumatol.

[12] Charnock D, Shepperd S, Needham G, Gann R (1999). DISCERN: an instrument for judging the quality of written consumer health information on treatment choices. J Epidemiol Community Health.

[13] Jung MJ, Seo MS (2022). Assessment of reliability and information quality of YouTube videos about root canal treatment after 2016. BMC Oral Health.

[14] Caloway C, Hersh CJ, Baars R, Sally S, Diercks G, Hartnick CJ (2019). Association of feeding evaluation with frenotomy rates in infants with breastfeeding difficulties. JAMA Otolaryngol Head Neck Surg.

[15] Dhir S, Landau BP, Edemobi S, Meyer AK, Durr ML (2022). Survey of pediatric otolaryngology frenotomy practice patterns. Laryngoscope.

[16] Naimer SA, Israel A, Gabbay A (2021). Significance of the tethered maxillary frenulum: a questionnaire-based observational cohort study. Clin Exp Pediatr.

[17] Ceremello PJ (1953). The superior labial frenum and the midline diastema and their relation to growth and development of the oral structures. Am J Orthod.

[18] Kotlow L (2011). Infant reflux and aerophagia associated with the maxillary lip-tie and ankyloglossia. Clin Lactation.

[19] Larrain M, Stevenson EG (2022). Controversy over tongue-tie: divisions in the community of healthcare professionals. Med Anthropol.

[20] Razdan R, Callaham S, Saggio R, Chafin M, Carr MM (2020). Maxillary frenulum in newborns: association with breastfeeding. Otolaryngol Head Neck Surg.

[21] Freeman CG, Ohlstein JF, Rossi NA, McIntire JB, Neve LD, Daram S, Pine HS (2022). Labial frenotomy for symptomatic isolated upper lip tie. Cureus.

[22] Wu V, Lee DJ, Vescan A, Lee JM (2022). Evaluating YouTube as a source of patient information for functional endoscopic sinus surgery. Ear Nose Throat J.

[23] Biggs TC, Bird JH, Harries PG, Salib RJ (2013). YouTube as a source of information on rhinosinusitis: the good, the bad and the ugly. J Laryngol Otol.

[24] Thomas C, Westwood J, Butt GF (2021). Qualitative assessment of YouTube videos as a source of patient information for cochlear implant surgery. J Laryngol Otol.

